# Effect of strontium inclusion on the bioactivity of phosphate-based glasses

**DOI:** 10.1007/s10853-017-1155-x

**Published:** 2017-05-11

**Authors:** J. K. Christie, N. H. de Leeuw

**Affiliations:** 1 Department of Materials, Loughborough University, Loughborough, LE11 3TU UK; 2 School of Chemistry, Cardiff University, Main Building, Park Place, Cardiff, CF10 3AT UK; 3 Department of Chemistry, University College London, 20 Gordon Street, London, WC1H 0AJ UK

## Abstract

We have conducted first-principles and classical molecular dynamics simulations of various compositions of strontium-containing phosphate glasses, to understand how strontium incorporation will change the glasses’ activity when implanted into the body (bioactivity). To perform the classical simulations, we have developed a new interatomic potential, which takes account of the polarizability of the oxygen ions. The Sr–O bond length is ∼2.44–2.49 Å, and the coordination number is 7.5–7.8. The Q^*n*^ distribution and network connectivity were roughly constant for these compositions. Sr bonds to a similar number of phosphate chains as Ca does; based on our previous work (Christie et al. in J Phys Chem B 117:10652, 2013), this implies that SrO ↔ CaO substitution will barely change the dissolution rate of these glasses and that the bioactivity will remain essentially constant. Strontium could therefore be incorporated into phosphate glass for biomedical applications.

## Introduction

Strontium is of profound importance in biomedical science [1], particularly because of its effect on bone growth. Strontium is naturally occurring in the human body, although at very low quantities, where it acts as a “bone seeker” like calcium, to the point that ∼99% of the body’s strontium is found in bone. Strontium is known to act synergistically with calcium to promote osteoblastic bone formation as well as inhibiting osteoclastic bone resorption [2]. As a result, strontium ranelate is an approved treatment for osteoporosis in postmenopausal women [3–6], who have an increased prevalence of the disease, whereas strontium malonate is currently undergoing clinical trials. Strontium intake also leads to greater deposition of calcium in bone [1]. Other medical uses of strontium include exploiting its bactericidal nature for injectable bone cement [7] and as an adjunct to radiotherapy [8].

In recent years, there has been substantial interest in incorporating strontium into other therapeutic materials which are implanted into the body. The dissolution products of many such materials are also known to promote new bone growth [9, 10]. Experimental [11, 12] and computational [13, 14] studies on strontium-containing bioglass have shown that the effect of strontium inclusion on the atomic structure is rather small, even for quite large (≤25%) concentrations of SrO [15]. However, the inclusion of Sr decreases bioactivity by decreasing the apatite-forming ability and dissolution rate [12] of bioglass.

Phosphate-based bioactive glasses (PBG) are also of considerable medical use, particularly because their dissolution rate is much faster than that of silicate-based glasses, and this rate can be tuned over several orders of magnitude by changes in the glass composition [9, 16]. This allows for the possibility of delivery of therapeutic ions to a localised site in the body at an appropriate rate, for the control of bone growth [10] among other treatments. It is natural therefore to ask whether Sr’s osteogenic properties could be combined with the favourable dissolution behaviour of PBG. To do this, however, we need to know the effect of strontium inclusion on the structure and degradation properties of PBG.

When Sr^2+^ was incorporated into (P_2_O_5_)_50_–(Na_2_O)_20_–(CaO)_30_ glasses, a disproportionation of *Q* ^2^ phosphorous atoms was seen, decreasing their prevalence and increasing the amount of *Q* ^1^ and *Q* ^3^ species [17]. Unexpectedly, the glass degradation rate increased with the addition of 1 mol% SrO (in place of Na_2_O) and then decreased with the addition of 3–5 mol% SrO, but it seems likely that this is due to varying phosphate contents (Table 4, Ref. [17]) in the prepared Sr-containing glasses, rather than any effect of the Sr itself. However, SrO substitution for CaO in Ti-containing ternary metaphosphate glasses seemed to show similar properties, with an increase by an order of magnitude in the degradation rate going from Sr-free to 1 mol% SrO, and then a slight decrease for SrO contents up to 5 mol%, with similar trends exhibited for ion release [18, 19]. The Sr-containing glasses were also found to be biocompatible, with excellent cell attachment and growth properties.

It is clear that the precise effect of strontium inclusion on phosphate-based bioactive glasses is not fully understood. If Sr-containing PBGs are to be used for the controlled release of therapeutic ions, then the connections between the glass composition, atomic structure and macroscopic properties such as degradation rate and cell adhesion must be understood, and computer simulation offers a route to achieve this goal. Molecular dynamics (MD) simulations provide the atomic structure of the glass, as well as enabling computation of large-scale materials properties. We and others have previously used MD simulations to elucidate the connection between atomic structure and dissolution behaviour of a variety of silicate [20–27] and phosphate glasses [28–30] intended for implantation, including strontium-containing silicate glass [13, 14]. However, there have been no simulations of Sr in phosphate glass.

In MD, the positions of the atoms evolve under Newton’s laws of motion. First-principles MD relies on a quantum–mechanical description of the interatomic forces, provided here through density functional theory (DFT). Although very accurate, the computational expense of such simulations is very high, and they are therefore limited to small models (a few hundreds of atoms) and short timescales. In classical MD, the interatomic forces are approximated by an empirical potential which is easier to compute, permitting the simulation of much larger models for longer timescales, but at the cost of introducing approximations and losing information on the electronic structure. It is therefore vital to derive a potential that is as accurate as possible. Previous simulations of Sr-containing silicate glasses [13, 14] have used a rigid-ion interatomic potential, in which the ions have partial charges and the polarizability of the ions is not included. Although this can often give satisfactory agreement with experimental data [22], it has been shown [31] that using a potential which incorporates the polarizability of the oxygen atoms, typically using the shell model, provides a more accurate representation of the medium-range structure of glasses, particularly the network connectivity and *Q* ^*n*^ distribution. The network connectivity, *Q* ^*n*^ distribution [21] and other medium-range structural motifs [28] are known to affect the bioactivity substantially. It is therefore essential that in any simulation, they are described correctly. We have therefore developed a polarizable potential for strontium, which is compatible with other potentials [32], and will offer an accurate description of the relevant structures and properties.

We have shown [28] that for polyphosphate (<50 mol% P_2_O_5_) glasses, in which the PO_4_ phosphate tetrahedra form finite-length unbranched chains, the dissolution rate is largely controlled by the interactions between these phosphate chains and the network-modifying cations (e.g., Na, Ca) to which they are bound. Ca binds to more chains than Na, and this strongly contributes to the known decrease in degradation rate [9] when Ca is substituted for Na, by strengthening the glass network. Since these compositions are commonly used [9], a full description of the effect of strontium inclusion on the dissolution of these glasses must therefore include its bonding to these chains.

In this work, we use classical and first-principles molecular dynamics to simulate several compositions of Sr-containing phosphate-based glasses. Following a description of the construction and validation of the new strontium potential, we describe the short-range structural environment around strontium, in terms of bond lengths, coordination environment and bond angles, before moving on to discuss the effect of strontium inclusion on the *Q* ^*n*^ distribution and network connectivity. We conclude with a discussion of strontium’s effect on the suitability of the glass for biomedical implantation.

## Development of a strontium interatomic potential

We sought to develop an accurate interatomic potential to take account of the inclusion of strontium into the glass structure. In our classical MD simulations, the interatomic interactions are described by the Born model of solids using full-charge pair potentials. In addition to the electrostatic interactions between ions, the short-range interactions are modelled by the Buckingham potential $V_{ij} \,\left( r \right) = A_{ij} \;\exp \left( {{{ - r} \mathord{\left/ {\vphantom {{ - r} {\rho_{ij} }}} \right. \kern-0pt} {\rho_{ij} }}} \right) - {{C_{ij} } \mathord{\left/ {\vphantom {{C_{ij} } {r^{6} }}} \right. \kern-0pt} {r^{6} }}$, where *r* is the interatomic distance between atoms of species *i* and *j* and $A_{ij}$, $\rho_{ij}$ and $C_{ij}$ are the parameters of the model. Our criteria were to develop a potential compatible with previous polarizable potentials developed for both phosphate [32] and silicate [33] glasses, so that it could be used to model the strontium environment in both types of glasses. (This work studies only phosphate glasses; Sr in silicate glasses will be the subject of future work.)

The GULP code [34] was used to fit the Sr–Os Buckingham potential to reproduce the crystal structures of SrO [35], SrSiO_3_ [36], Sr_2_SiO_4_ [37] and Sr_3_(PO_4_)_2_ [38], i.e., including oxide, silicate and phosphate environments. The other potential interactions were held constant to the values (Table 1) which have previously been used to model a wide range of silicate and phosphate glasses [32, 39]. The final potential parameters are listed in Table 1, and the lattice parameters of the crystalline structures are given in Table 2.

**Table 1:** The interatomic potential parameters used in this work

Pairs	*A* (eV)	*ρ* (Å)	*C* (eV Å^6^)
$$ V = A_{ij} \;\exp \left( {{{ - r} \mathord{\left/ {\vphantom {{ - r} {\rho_{ij} }}} \right. \kern-0pt} {\rho_{ij} }}} \right) - {{C_{ij} } \mathord{\left/ {\vphantom {{C_{ij} } {r^{6} }}} \right. \kern-0pt} {r^{6} }} $$			
Sr–Os	2472.714957	0.314337	0.121766
Na–Os [33]	56465.3453	0.193931	0.0
Ca–Os [33]	2152.3566	0.309227	0.09944
P–Os [32]	1020.00	0.343220	0.03
Os*–*Os [39]	22764.30	0.149	27.88

Triplets	*k*_3b_ (eV rad^−2^)	*θ*_0_ (°)
$$ k_{{3{\text{b}}}} \,\left( {\theta_{jik} - \theta_{0} } \right)^{2} $$		
Os–P–Os [32]	3.3588	109.47
P–Os–P [32]	7.6346	141.1793
Os–Si–Os [33]	2.097	109.47

Core-shell	*k*_cs_	*q* (Oc)
$$ k_{\text{cs}} \left( {{\text{r}}\left( {\text{Oc}} \right) - \text{r} \left( {\text{Os}} \right)} \right)^{2} $$		
Oc–Os [39]	74.92	+0.8482

**Table 2:** The calculated and experimental lattice parameters for strontium crystals using the interatomic potentials developed in this work

Material	Parameter	Calc.	Exp.	Diff.
Sr_3_(PO_4_)_2_	*a*, *b*	5.491 Å	5.378 Å	+2.1%
	*c*	19.561 Å	19.754 Å	−1.0%
	*α*, *β*		90.0°	
	*γ*		120.0°	
SrO	*a*, *b*, *c*	5.018 Å	5.156 Å	−2.7%
	*α*, *β*, *γ*		90.0°	
SrSiO_3_	*a*	12.286 Å	12.333 Å	−0.4%
	*b*	7.147 Å	7.146 Å	+0.0%
	*c*	10.960 Å	10.885 Å	+0.7%
	*α*, *γ*		90.0°	
	*β*	110.87°	111.57°	−0.6%
Sr_2_SiO_4_	*a*	9.756 Å	9.767 Å	−0.1%
	*b*	7.093 Å	7.084 Å	+0.1%
	*c*	11.061 Å	11.059 Å	+0.0%
	*α*, *γ*	90.0°		
	*β*	148.89°	149.24°	−0.2%

It is clear that the Sr–Os potential works well in diverse oxide environments, as none of the errors in the lattice parameters or bond angles is larger than 2.7%, and most are considerably smaller. Moreover, the potential performs better, in the sense that the errors are smaller, in the silicate and phosphate environments in which it is intended to be used.

## Methods

Our interatomic potential was used in the DLPOLY code [40] to conduct classical MD simulations of a variety of compositions of strontium-containing phosphate glasses based on compositions with 45 mol% P_2_O_5_, 25 mol% Na_2_O and 30 mol% CaO, which have been studied both experimentally [17] and in simulation [28]. SrO was substituted both for CaO (the other alkaline earth oxide) and for Na_2_O to facilitate a comparison to experimental data. The precise compositions and densities studied are given in Table 3. Classical MD was performed on the compositions with 1 and 3 mol% SrO, and for each of these compositions, about 3000 atoms were placed independently and randomly into a cubic periodic box to give the appropriate density (Table 3) subject only to the constraint that atoms not be placed closer than 85–90% of their expected interatomic distances to avoid unphysical starting configurations. Three independent configurations were prepared for each of these compositions. Following an instantaneous relaxation at zero temperature, the classical models were run in an NVT ensemble for 50 ps at 2500 K, before being cooled in a series of runs in NVT ensembles of length 50 ps, decreasing the temperature by 200 K in each run, at a cooling rate of 4 K/ps. The model was then run in an NVT ensemble at 300 K for 200 ps, the final two-thirds of which formed the production run. Unless otherwise stated, all data are averaged over snapshots taken from this production run and over the three independent models. The timestep was 0.2 fs, and the potentials were truncated at 8 Å. The Ewald cut-off was 12 Å.

**Table 3:** The glass compositions, densities and numbers of atoms studied in this work

Name	Mol%				Density	Numbers of atoms				
	P_2_O_5_	Na_2_O	CaO	SrO	(g/cm^3^)	O	P	Na	Ca	Sr
P45NSr1	45.0	24.0	30.0	1.0	2.57	1872	602	320	200	7
P45NSr3	45.0	22.0	30.0	3.0	2.58	1879	604	296	201	20
P45CSr1	45.0	25.0	29.0	1.0	2.57	1867	600	334	193	7
P45CSr3	45.0	25.0	27.0	3.0	2.58	1867	600	334	180	20
P45NSr10	45.0	15.0	30.0	10.0	2.64	93	30	10	10	3
P45CSr10	45.0	25.0	20.0	10.0	2.64	93	30	16	7	3

First-principles molecular dynamics, in which the interatomic forces are derived from a quantum–mechanical representation of the electronic structure, was performed on the compositions with 10 mol% SrO (Table 3). Due to the greater computational expense of these simulations, they can only be performed on relatively small models and for shorter timescales. In order to ensure adequate sampling of the strontium environment, therefore, two compositions with this rather high SrO content were modelled. The CP2K code [41] was used to perform Born–Oppenheimer molecular dynamics on these systems. For each composition, about 150 atoms were placed independently and randomly into a cubic periodic box to give the appropriate density (Table 3) again subject to the constraint that atoms not be placed closer than 85–90% of their expected interatomic distances. The models were then run in an NVT ensemble at 2500 K for 20 ps, before being cooled in a series of runs in NVT ensembles of length 10 ps, decreasing the temperature by about 300 K in each run, corresponding to a cooling rate of ∼30 K/ps. The models were then run in an NVT ensemble at 300 K for ∼40–50 ps, the final two-thirds of which formed the production run. Unless otherwise stated, all data are averaged over snapshots taken from this production run. The plane-wave cut-off was 600 eV, and the timestep was 1.0 fs. PBE [42] exchange–correlation functionals in the generalised gradient approximation (GGA) were used.

Although the cooling rates for both classical and first-principles models are much faster than those used experimentally, models prepared using these methodologies have given structures in agreement with experiment for silicate [20, 43–45] and phosphate glasses [28, 46], including for compositions intended for implantation.

## Results

The relationships between the structure and bioactivity of strontium-free phosphate-based glasses have been discussed elsewhere [9, 16, 28]. Therefore, in this work, we concentrate on characterising the strontium environment.

Table 4 shows the bond lengths and coordination numbers for each composition. The Sr–O bond lengths for the classical models are between 2.44 and 2.49 Å; those for the first-principles models are slightly longer at 2.55–2.57 Å. The generalised gradient approximation (GGA) [42] which we have used is known to underbind certain bonds, which would lead to longer bond lengths, and it seems that this is happening here.

**Table 4:** The Sr–O bond lengths and coordination numbers (using a cut-off of 3.5 Å) for the compositions studied

Name	Bond length (Å)	Coordination number
P45NSr1	(2.45 ± 0.04)	7.8 ± 0.5
P45NSr3	(2.44 ± 0.02)	7.6 ± 0.1
P45CSr1	(2.49 ± 0.04)	7.5 ± 0.5
P45CSr3	(2.46 ± 0.02)	7.5 ± 0.2
P45NSr10	2.55	8.0
P45CSr10	2.57	8.7

Errors quoted are SDs from the independent configurations

**Figure 1:**
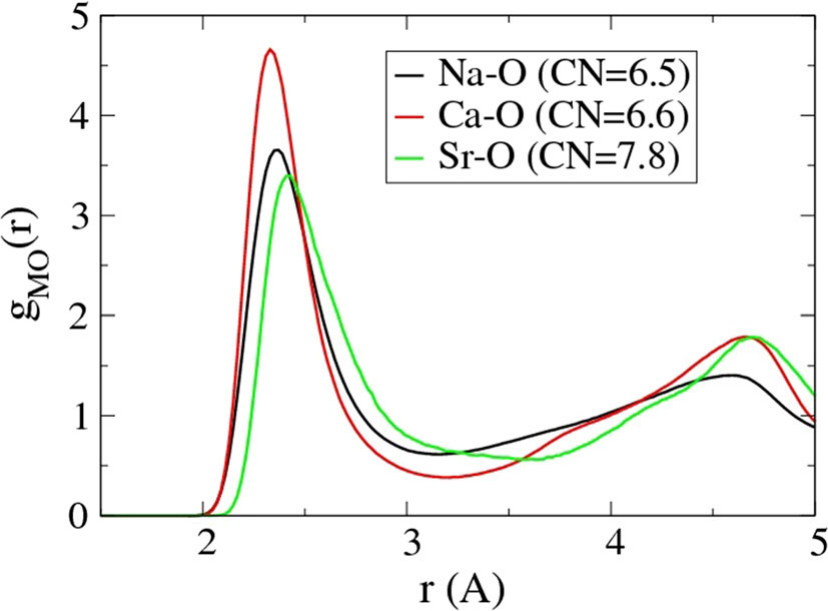
The partial pair-correlation functions *g*_NaO_(*r*), *g*_CaO_(*r*) and *g*_SrO_(*r*) for one model of P45CSr3, as a representative composition. Coordination numbers are included in the legend; the cut-offs are 3.2 Å for Na–O and Ca–O and 3.5 Å for Sr–O

Coordination numbers vary from 7.5 to 7.8 for the classical models and are slightly larger for the first-principles models, which is likely due to the longer bond lengths as discussed above. Both the Sr–O bond lengths and coordination numbers are larger than the Na–O and Ca–O bond lengths and coordination numbers, which has profound effects on the bioactivity, as explained later. Figure 1 shows partial pair-correlation functions for the three metal–oxygen pairs in a representative composition, which also shows clearly the larger cut-off needed to define the first coordination shell of strontium.

Experimental estimates of the Sr–O bond length and coordination number have not been performed in phosphate glasses, to the best of our knowledge, although the bond lengths we find are in good agreement with those from strontium borosilicate glass (2.52 Å) [47] and strontium tin silicate glass (2.59–2.64 Å) [48]. The Sr–O coordination number was between four and five in the borosilicate glass, in contradiction to our results, but it was between seven and eight for the tin silicate glass and around seven in other computational studies [13, 14].

Some oxygen atoms are bound to two phosphorus atoms, bridging two PO_4_ tetrahedra; these are known as bridging oxygen (BO) atoms. Modifier ions prefer to bond to non-bridging oxygen (NBO) atoms, which typically carry a greater charge. When there is more than one modifier atom in a glass composition, they compete for these NBOs, leading to different amounts of bonding to BO and NBO atoms for different modifiers [49]. In our models, the amounts of NBO bound to each modifier are shown in Table 5. For all compositions and methodologies, Na has the smallest percentage of NBOs, and Ca the most, with Sr between the two.

**Table 5:** The percentage of non-bridging oxygen atoms in the first coordination shell of the modifier atoms for all compositions

Name	NBO neighbours		
	Na (%)	Sr (%)	Ca (%)
P45NSr1	(80 ± 1)	(86 ± 4)	(93 ± 1)
P45NSr3	(80 ± 1)	(87 ± 2)	(93 ± 1)
P45CSr1	(80 ± 0)	(91 ± 1)	(94 ± 0)
P45CSr3	(80 ± 0)	(87 ± 1)	(94 ± 1)
P45NSr10	86	89	95
P45CSr10	83	88	92

Errors quoted are SDs from the independent configurations

**Figure 2:**
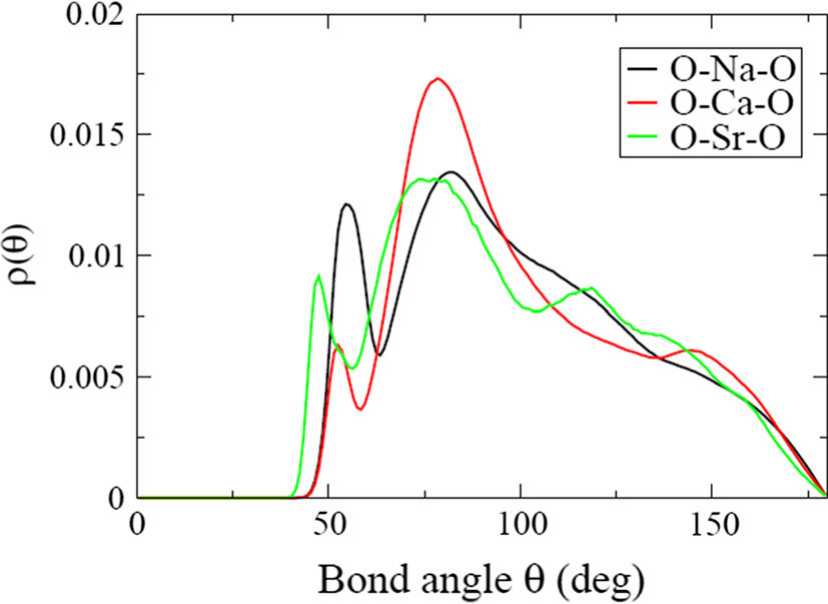
The O–Na–O (*black*), O–Ca–O (*red*) and O–Sr–O (*green*) bond-angle distributions for one model of P45NSr3, as a representative composition. The cut-offs are 3.2 Å for Na–O and Ca–O and 3.5 Å for Sr–O

The bond-angle distribution around the modifier atoms also reveals something of their local environments. As before, there is little difference between compositions, at least for those simulated with the same methodology. In Fig. 2, the O–M–O bond-angle distribution, where M = Na, Ca or Sr, is displayed for a representative composition. For each distribution, a very broad distribution of bond angles is seen, reflecting the range of coordination environments and the disorder inherent in the structure. The O–Sr–O bond-angle distribution extends to more acute bond angles than the other two, reflecting strontium’s higher coordination number and the need for more acute bond angles to accommodate seven or even eight oxygen atoms in the coordination shell. All three distributions have a broad peak at about 90°, corresponding to a distorted octahedral coordination shell typical of six-coordinated atoms. For O–Sr–O, this peak is at more acute angles because of the greater number of higher-coordinated atoms.

One of the key parameters which controls the bioactivity of glasses implanted into the human body is the network connectivity [21], defined as the average number of bridging oxygen (BO) atoms per phosphorus atom. CaO ↔ SrO substitution is thought unlikely to change the network connectivity or *Q* ^*n*^ distribution (*n* is the number of BOs) because the ratio of O atoms to P atoms remains constant. We computed the *Q* ^*n*^ distribution for the compositions prepared with classical MD to find that they were all almost identical, with 26–27% *Q* ^1^, 68–70% *Q* ^2^, and 4% *Q* ^3^ for a network connectivity of 1.77–1.78.

In previous work [28], we and others have identified that one of the most relevant structural features for glasses with <50 mol% P_2_O_5_ (equivalently, phosphate glasses with a network connectivity less than 2) is the extent to which the modifier ions bind to different numbers of phosphate chains. We are able to compute this for the three different modifier ions present in our compositions here, and these results are presented in Table 6. There is a clear difference between the 3.2–3.3 phosphate chains bound to the singly charged sodium ion, and the 3.9 or more chains bound to the doubly charged calcium and strontium ions, as we identified previously for Sr-free glasses [28]. Sr seems to have a similar number—perhaps very slightly larger—of chains bound to it as are bound to Ca.

**Table 6:** The average number of fragments bound to each Na, Ca or Sr atom for the glass compositions studied

	No. of fragments		
	Na	Sr	Ca
P45NSr1	3.2 ± 0.1	3.9 ± 0.2	3.9 ± 0.1
P45NSr3	3.2 ± 0.1	4.0 ± 0.1	3.9 ± 0.0
P45CSr1	3.2 ± 0.0	4.1 ± 0.5	3.9 ± 0.1
P45CSr3	3.3 ± 0.0	4.1 ± 0.2	4.0 ± 0.1

Errors quoted are SDs from the independent configurations

## Discussion

We have investigated the effects caused by the incorporation of strontium, which has osteogenic and other useful medical properties, into bioactive phosphate glasses, which have a tunable dissolution rate. We have focussed on understanding how strontium changes the atomic structure, and how this is likely to affect the bioactivity.

To do this, we had to develop an accurate potential to represent the interatomic forces involving strontium. The structural features in glasses which most affect the bioactivity are known to occur at the medium-range length scales, i.e., the network connectivity, *Q* ^*n*^ distribution [21] and phosphate-chain to network-modifier bonding [28]. It is also known that to describe this medium-range structure well using classical molecular dynamics simulations requires a polarizable potential [31]. In order to maximise its utility, therefore, we have developed a polarizable potential that is compatible with previous work in both phosphate and silicate glasses, and we have shown that this potential model represented the crystal structures of strontium oxide, silicates and phosphate, rather well.

Using this new potential, we have shown that the Sr–O bond length is ∼2.44–2.49 Å and the coordination number is 7.5–7.8; both of these quantities are larger than for Na–O or Ca–O, which have typical coordination numbers of less than seven. Although all three modifier ions prefer to bond to non-bridging oxygen atoms, the extent to which each of them does so depends on their field strength [24]. Sodium has the lowest field strength and the lowest percentage of NBOs in its first coordination shell and calcium has the highest field strength and the largest percentage of NBOs in its first coordination shell, while strontium is intermediate between the two. This relationship between field strength and percentage of NBO’s takes no account of the different coordination numbers of the three ions. Similarly, the amount of intra-tetrahedral bonding, where two oxygen atoms from one PO_4_ tetrahedron are bonded to the same modifier atom, varies, being most pronounced in Na, and least visible in Ca, as can be seen by the height of the peak (proportional to the amount of intra-tetrahedral bonding) at about 60° in the O–(Na, Ca, Sr)–O bond-angle distributions.

The network connectivity and *Q* ^*n*^ distribution are essentially unaffected by the incorporation of strontium. This is to be expected, certainly for the network connectivity, which depends on the ratio of the number of oxygen atoms to the number of phosphorus atoms, which does not change on either SrO ↔ CaO or SrO ↔ Na_2_O substitution. The *Q* ^*n*^ distribution might change, but the amounts of Sr incorporated here are small, and so the associated change is small.

As for other glasses with similar phosphate contents [28], the dissolution rate of these glasses will be controlled by the different bonding of the modifier ions to the surrounding phosphate chains, which we have characterised. Despite the differences in their local environments in terms of coordination numbers and percentage of non-bridging oxygen atoms in their coordination shell, strontium and calcium bond to a very similar number of fragments. We predict, therefore, that SrO ↔ CaO will not affect the dissolution rate substantially. Substituting SrO for Na_2_O will affect the dissolution rate, decreasing it with increasing Sr content, as occurs when CaO substitutes Na_2_O. Based on this prediction, we expect that there will be no structural obstacles to the incorporation of strontium into phosphate-based bioactive glasses for biomedical implantation, and indeed, it may be possible to fine-tune the dissolution rate through careful analysis of the SrO/CaO ratio.

## Conclusion

We have conducted first-principles and classical molecular dynamics simulations of various compositions of strontium-containing phosphate glasses, to understand how strontium incorporation will change the glasses’ activity when implanted into the body (bioactivity). To perform the classical simulations, we have developed a new interatomic potential, which takes account of the polarizability of the oxygen ions. Our previous work [28–30] on the dissolution of phosphate glasses had shown us that the number of phosphate chains chemically bound to each network modifier is a key parameter to understand the associated effect on the dissolution. We have found that strontium bonds to a similar number of phosphate chains as calcium does. The implication is that SrO ↔ CaO substitution will cause little or no change in the dissolution rate of these glasses and that the bioactivity will remain essentially constant. We conclude, therefore, that strontium could be incorporated into phosphate glass for biomedical applications.
